# IL‐1 receptor antagonist anakinra downregulates inflammatory cytokines during renal normothermic machine perfusion: Preliminary results

**DOI:** 10.1111/aor.14909

**Published:** 2024-11-20

**Authors:** Sarah A. Hosgood, Tom Moore, Alex Walker, Michael L. Nicholson

**Affiliations:** ^1^ Department of Surgery Addenbrooke's Hospital, University of Cambridge Cambridge UK

**Keywords:** anakinra, normothermic machine perfusion, renal transplantation

## Abstract

**Background:**

The interleukin 1 (IL‐1) cytokine group plays a key role in sterile inflammation and may be an important target for transplant‐related renal injury. This study examined the effects of anakinra, a non‐specific IL‐1 receptor antagonist, administered during normothermic machine perfusion (NMP) of porcine kidneys.

**Method:**

Paired porcine kidneys (*n* = 5 pairs) underwent 15 min of warm ischemia plus 2 h of static cold storage in ice. Kidneys were then perfused with autologous whole blood using an ex vivo NMP platform. Kidneys were randomly allocated to receive anakinra or vehicle administered at the start of NMP. Cortical biopsies were collected at baseline before ischemic injury and at the end of NMP. Functional parameters were recorded and calculated, and inflammatory markers were measured by qPCR and ELISA techniques.

**Results:**

During NMP, there were no statistically significant differences in renal blood flow, urine output, creatinine clearance or fractional excretion of sodium in the anakinra and control groups. The administration of anakinra significantly downregulated transcriptional expression of IL‐6, Fas ligand and intercellular adhesion molecule 1 (*p* = 0.029, 0.029, 0.028, respectively).

**Conclusion:**

Anakinra, an IL‐1 receptor blocker, successfully attenuated the downstream inflammatory and immune‐mediated response within the kidney during NMP.

## Graphical abstract

Anakinra, an IL‐1 receptor antagonist, attenuated the inflammatory response during normothermic machine perfusion (NMP) in porcine kidneys subjected to mild ischemic insult. The preliminary study provides insight into the underlying mechanisms of renal injury and indicates NMP has potential as a platform for the targeted delivery of treatments to kidneys pre‐transplantation.

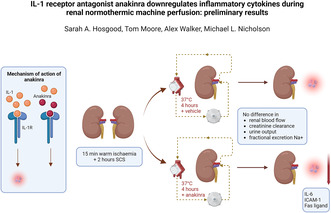

## 1 INTRODUCTION

Machine perfusion technologies are being introduced in transplantation to increase organ utilization and improve graft function. The restoration of circulation and metabolism with an oxygenated blood‐based solution during normothermic machine perfusion (NMP) may be particularly beneficial for the administration of targeted treatments to ameliorate injury prior to transplantation. Although there is an abundance of research investigating different therapies,^1^, ^2^ there has been little development in the clinical application of NMP as an interventional treatment device.

One potential target for intervention is the inflammasome, interleukin 1α (IL‐1α)/interleukin 1β (IL‐1β)/IL‐1 receptor 1 (IL‐1R1) system, a key component of inflammation in renal ischemia reperfusion injury.^3^, ^4^ IL‐1α and IL‐1β bind to IL‐1R1 to activate transcription factors, including nuclear factor kappa B (NF‐κB), leading to the production of pro‐inflammatory cytokines and the initiation of apoptosis.^3^ Anakinra is a recombinant human IL‐1 receptor antagonist (IL‐1RA) consisting of 153 amino acid residues. It competitively inhibits binding of IL‐1α and IL‐1β to IL‐1R1, thus preventing the cascade of sterile inflammation and the assembly of the NLRP3 inflammasome. Anakinra is licensed for the treatment of autoimmune diseases, including rheumatoid arthritis, but is also used to treat a wide range of other diseases.^5^ The mechanism of action of anakinra suggests that it may have a role in ameliorating transplant‐related renal injury.^6^

The aim of this study was to determine the physiological and molecular effects of IL‐1R blockade using anakinra during ex vivo NMP of porcine kidneys.

## 2 METHODS

### 2.1 Study design

Porcine kidneys were subjected to 15 min of in situ warm ischemia followed by 2 h of static cold storage in ice and then a 4 h period of NMP using a blood‐based perfusate. Pairs of kidneys from the same animal (*n* = 10 kidneys) were randomly allocated to receive anakinra (50 mg) or a vehicle control (0.5 mL of 0.9% sodium chloride) delivered as an arterial bolus at the start of the 4 h period of NMP. The anakinra dosage was selected to ensure maximum saturation in this ex vivo kidney model.^7^

### 2.2 Kidney retrieval

Approval to carry out the research study at a licensed commercial research establishment in the UK was granted under the Home Office “The Animals” (Scientific Procedures) Act 1986. In accordance with relevant guidelines and regulations, five landrace cross female pigs weighing 40–45 kg underwent general anesthesia and midline laparotomy (study number YH41HK). Both renal pedicles were exposed and the kidneys dissected. A bolus injection of 25 000 IU heparin was given 5 min before the renal artery and renal veins were ligated for 15 min to provide a short period of warm ischemic injury. After the warm ischemic interval, the kidneys were removed and flushed with 500 mL of ice‐cold University of Wisconsin (UW) preservation solution at a hydrostatic pressure of 100 cmH_2_O. Kidneys were then placed in bags containing ice‐cold UW solution for transportation to the perfusion laboratory. One liter of blood was collected from the donor animal's aorta into two citrate–phosphate‐dextrose‐adenine (CPDA) blood bags before the animal was euthanized with an overdose of barbiturate.

### 2.3 Ex vivo normothermic machine perfusion (NMP)

After removal from cold storage (2 h), the kidneys were weighed and prepared for NMP. The renal artery, vein, and ureter were cannulated; and the kidney was flushed with 200 mL of cold Ringer's solution to remove the preservation solution, which has a high potassium concentration.

NMP was carried out using a perfusion circuit based on cardiopulmonary bypass technology and extracorporeal membrane oxygenation using the Medtronic Bioconsole 560 as previously described.^8^ The ex vivo perfusion circuit was primed with 300 mL of Ringer's solution (Baxter Healthcare, Thetford, UK). Mannitol 2.5 g (Sigma‐Aldrich, UK), sodium bicarbonate 8.4% (15 mL) and 3000 IU of heparin were added to the circuit. Autologous whole blood (300 mL) was then added and recirculated to reach a temperature of 37.4°C. The blood‐based solution was circulated continually through the kidney via the renal artery at a mean arterial pressure of 85 mmHg. A nutrient solution (Synthamin 17 10%, Baxter Healthcare, Thetford, UK) with 15 mL of sodium bicarbonate 8.4% and 100 IU of insulin added (Actrapid) was infused at a rate of 20 mL/h, glucose 5% at a rate of 5 mL/h, and Ringer's solution was used to replace urine output mL for mL. The blood‐based perfusate was oxygenated via the membrane oxygenator incorporated into the circuit with a 95% oxygen/5% carbon dioxide flow rate of 0.1 L/min. Creatinine was infused into the arterial arm of the circuit to maintain a mean circulating concentration of 2000 μmol/L. NMP was carried out for 4 h and renal blood flow was recorded continuously throughout this period.

### 2.4 Sample collection

Samples of perfusate and urine were collected immediately before the start of NMP and hourly thereafter. Samples were sent for routine hematology and biochemical analysis including urea and electrolytes. Additional aliquots of perfusate and urine were stored for further analysis at −80°C. Creatinine clearance was calculated as the product of urine creatinine (μmol/L) and volume of urine (mL) divided by plasma creatinine (μmol/L). Fractional excretion of sodium (FENa) was calculated as the product of urinary sodium and plasma creatinine divided by the product of plasma sodium and urinary creatinine. Renal cortical biopsies were performed before the onset of the 15 min period of warm ischemia (baseline) and at the end of the 4 h NMP period. Samples were divided and either snap frozen in liquid nitrogen, fixed in RNAlater solution or fixed in 10% formalin.

### 2.5 Cytokines

Inflammatory cytokines IL‐6, IL‐1β, IL‐8, and tumor necrosis factor α (TNFα) were measured in the perfusate by ELISA at baseline (pre‐perfusion) and at the end of the 4 h of NMP. Cytokine levels were also measured in urine samples taken after 1, 3, and 4 h of NMP.

### 2.6 qPCR

qPCR was used to quantify the changes in gene expression in kidneys treated with anakinra compared with the paired control kidney. Biopsies were stored in RNAlater solution (Invitrogen RNAlater™ Soln) for fixing. The tissue was lysed (Precellys Lysing Kit MK28‐R) and RNA was extracted (Invitrogen PureLink™ RNA Mini Kit and Invitrogen TURBO DNA‐free™ Kit). The RNA was used to generate the cDNAs for each sample by reverse transcription (Applied Biosystems High Capacity cDNA Reverse Transcription Kit). Primers (Table S1) were designed using NCBI Primer Blast for angiopoietin 1, Fas ligand, hypoxanthine phosphoribosyltransferase 1 (HPRT1), intercellular adhesion molecule 1 (ICAM‐1), IL‐1β, IL‐6, and IL‐8. HPRT1, the housekeeping gene, encodes for the protein hypoxanthine‐guanine phosphoribosyltransferase (HGPRT), which is an enzyme involved in purine synthesis. Relative RT‐PCR was performed (Stratagene Mx3005P) using SYBR Green (Applied Biosystems *Power* SYBR® Green PCR Master Mix) on all kidney samples for each gene in triplicate. Cycling conditions are shown in Table S2. Analyses were performed using the 2^−ΔΔCt^ method between anakinra and control groups at 4 h of NMP and baseline in individual kidneys.

### 2.7 TUNEL staining

To examine the effect of anakinra on apoptosis, sections of paraffin‐embedded renal cortex, taken at baseline and after 4 h of NMP, were stained using the TUNEL technique and visualized using a fluorescence microscope. Ten different fields of view at ×40 magnification images were taken in 4′,6‐diamidino‐2‐phenylindole (DAPI) and fluorescein isothiocyanate (FITC) wavelength for each field of view and the number of positive cells was counted.

### 2.8 Statistics

The D'Agostino–Pearson normality test was used to determine whether the data were normally distributed. Values are presented as mean ± standard deviation or median and interquartile range as appropriate. Non‐parametric and parametric data were analyzed using the Wilcoxon matched‐pairs signed rank test or a paired *t*‐test respectively. For qPCR, data were plotted as mean and SEM. Statistical analysis was carried out using a non‐parametric paired *t*‐test. *p* ≤ 0.050 was considered statistically significant. Statistical analysis was performed using Microsoft Excel and GraphPad Prism 8 (GraphPad Software Inc., La Jolla, CA, USA). GraphPad software was also used to randomly assign five kidneys (one of each pair) into the two groups.

## 3 RESULTS

### 3.1 Anakinra had no effect on renal hemodynamics or function during NMP

**FIGURE 1:**
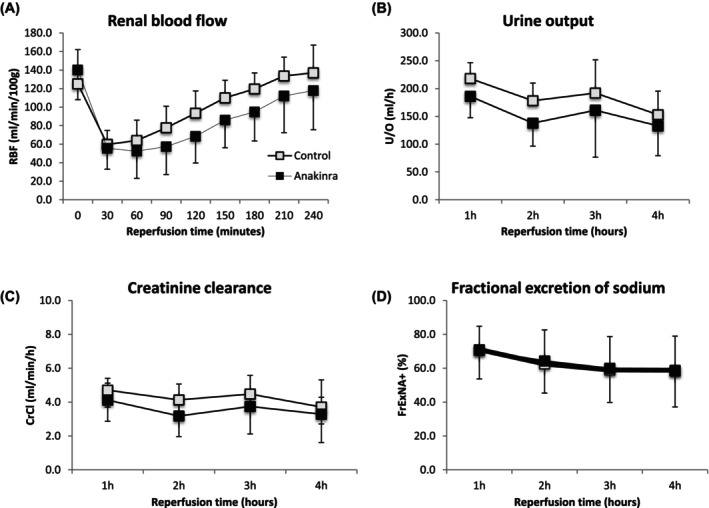
(A) Renal blood flow, (B) urine output, (C) creatinine clearance, and (D) fractional excretion of sodium in the control and anakinra groups during 4 h of reperfusion. Mean ± SD renal blood flow was plotted every 30 min; urine output, creatinine clearance, and fractional excretion of sodium plotted every hour.

To prevent the binding and activation of IL‐1, anakinra, an IL‐1RA, was administered to porcine kidneys during NMP. Anakinra did not change renal blood flow, urine production, creatinine clearance, or levels of fractional excretion of sodium compared with controls (Figure 1A–D; Table 1).

**TABLE 1:** Mean renal blood flow (RBF), creatinine clearance (CrCl), fractional excretion of sodium (Fr Ex Na+) and total urine output (U/O) in porcine kidneys treated with anakinra versus control during 4 h of reperfusion.

	Control	Anakinra	*p* value
RBF (mL/min/100 g)	102.3 ± 16.0	87.2 ± 26.5	0.271
CrCl (mL/min/100 g)	4.3 ± 0.9	3.6 ± 1.2	0.338
Fr Ex Na+ (%)	62.8 ± 18.2	63.3 ± 18.8	>0.999
Total U/O (mL)	741 ± 117	618 ± 194	0.193

### 3.2 Anakinra reduced renal cortical expression of IL‐6, ICAM‐1, and Fas ligand

**FIGURE 2:**
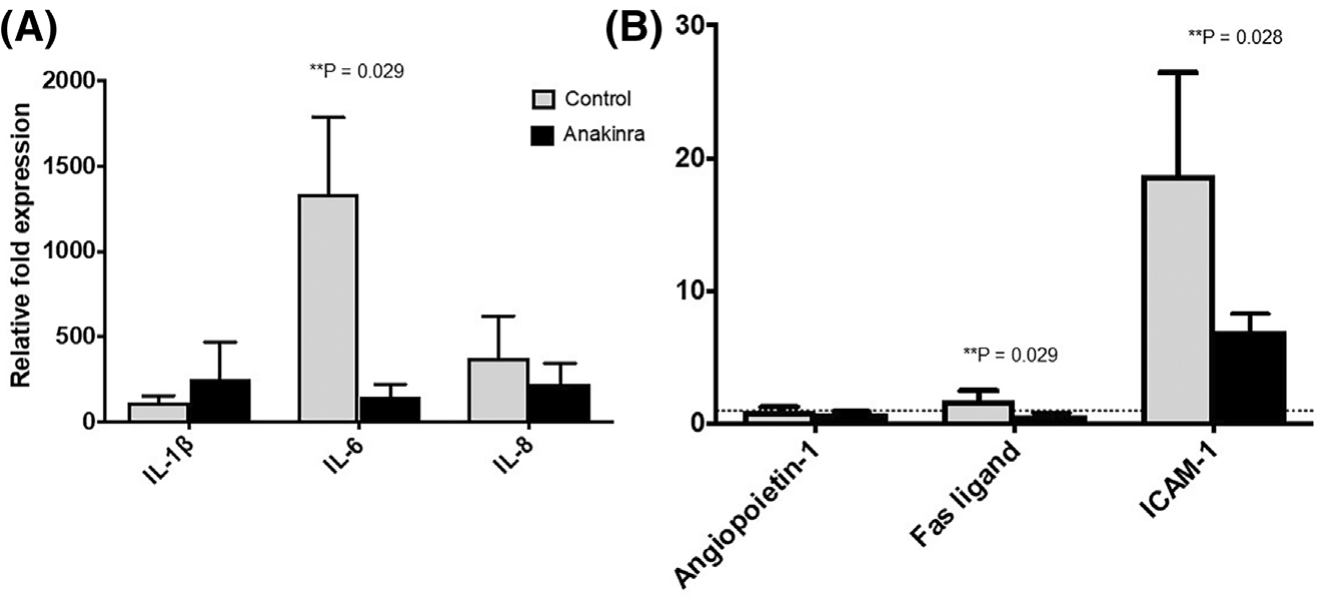
mRNA expression of (A) IL‐1β, IL‐6, and IL‐8 and (B) angiopoietin‐1, Fas ligand, and ICAM‐1 at 4 h reperfusion compared with baseline in the control and anakinra groups measured by qPCR. Anakinra significantly reduced the expression of IL‐6, Fas ligand and ICAM‐1 compared with control kidneys (**p = 0.029, 0.029, 0.028 respectively).

To determine the effects of anakinra on gene expression, baseline and end‐perfusion cortical biopsy samples were used to compare transcriptional expression of the inflammatory cytokines IL‐1β and IL‐6 and the chemokine IL‐8. The effect of anakinra on angiopoietin‐1, Fas ligand, and ICAM‐1 expression in the renal cortex was also measured. Fas ligand is a type‐II transmembrane protein that belongs to the TNF family; ICAM‐1 (CD54) is an endothelial and immune cell adhesion molecule that is induced by IL‐1 through the activation of NF‐κB; and angiopoietin‐1 is an endothelial‐specific growth factor that acts through a tyrosine kinase receptor to promote vascular integrity. Anakinra delivered during NMP significantly reduced renal cortical expression of IL‐6, Fas ligand, and ICAM‐1 compared with control kidneys (*p* = 0.029, 0.029, 0.028 respectively; Figure 2A,B). In contrast, anakinra had no significant effect on renal cortical expression of IL‐1β or IL‐8 (*p* > 0.05).

### 3.3 Anakinra levels in the NMP perfusate

**FIGURE 3:**
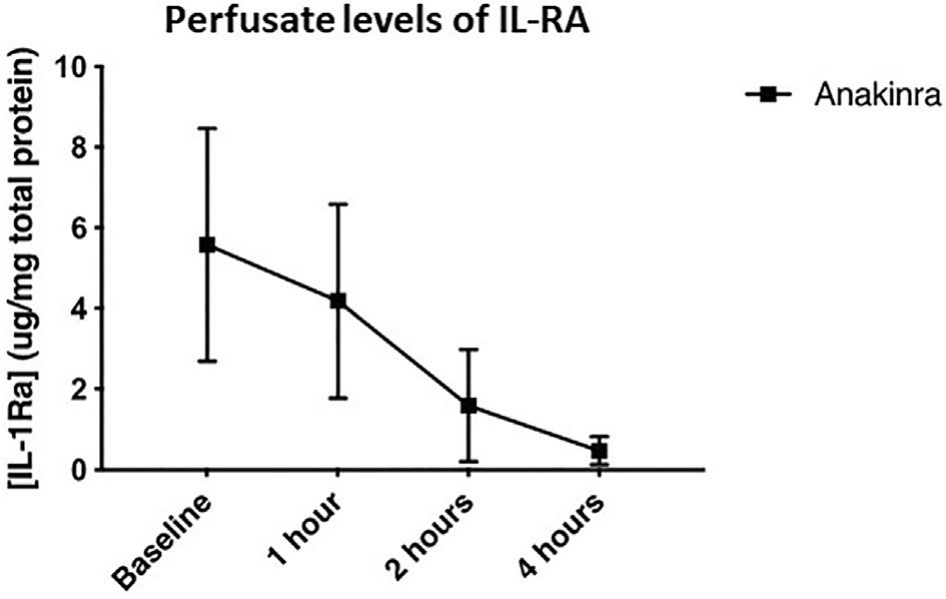
Mean ± SD levels of IL‐RA in the perfusate pre‐reperfusion and at 1, 2, 3, and 4 h during reperfusion measured by ELISA. Levels were undetectable in the control group.

Levels of circulating anakinra were measured in the perfusate by ELISA (Figure 3). IL‐1RA was only detectable in treated kidneys and decreased in an approximately linear manner over the 4 h of NMP. At 4 h IL‐1RA levels were negligible.

### 3.4 Anakinra reduced IL‐6 levels in the urine during NMP

**FIGURE 4:**
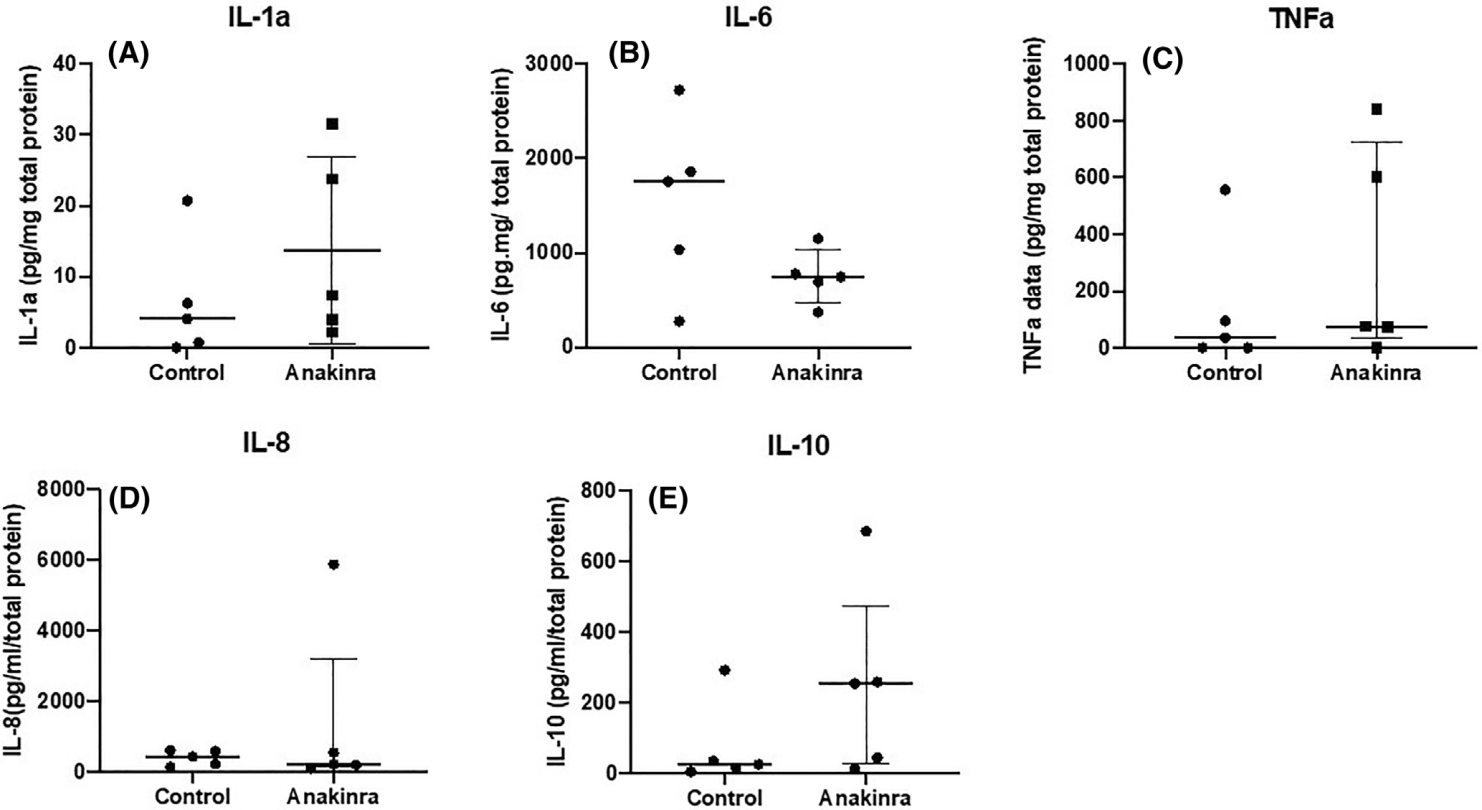
Mean ± SD levels of (A) IL‐1α, (B) IL‐6 and median and interquartile range of (C) TNFα, (D) IL‐8, and (E) IL‐10 measured in the perfusate in the control and anakinra groups after 4 h of reperfusion measured by ELISA. There was no significant difference in the levels between the groups (*p* > 0.05). Levels of IL‐1β were undetectable in both groups during reperfusion.

There were no statistically significant differences between perfusate levels of IL‐1α, IL‐6, TNFα, IL‐8, and IL‐10 in anakinra‐treated and control kidneys (Figure 4B–E; *p* > 0.050). Levels of IL‐1β were not detectable in the control or the anakinra‐treated kidneys (data not shown).

**FIGURE 5:**
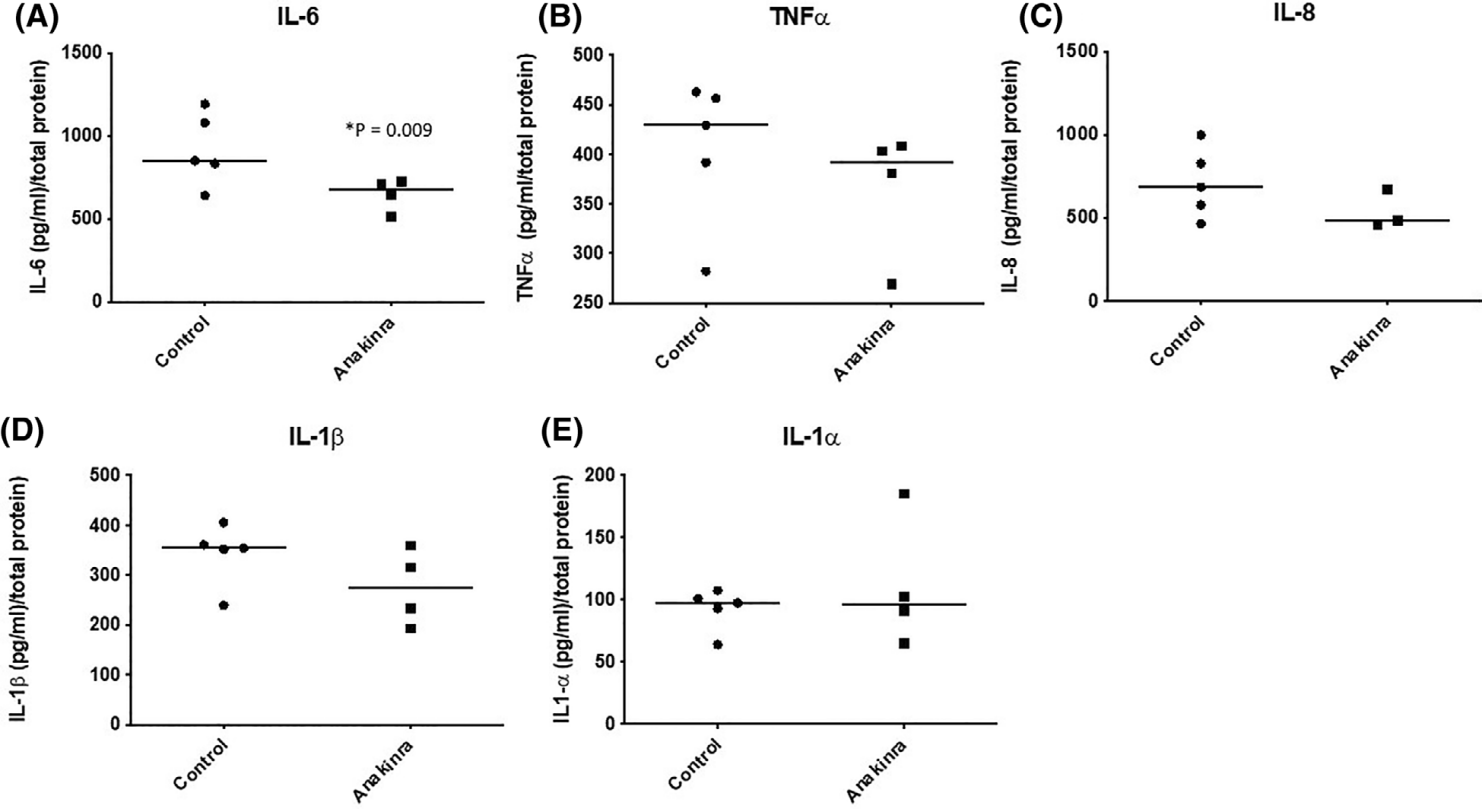
Mean ± SD levels of levels of (A) IL‐6, (B) TNFα, (C) IL‐8, (D) IL‐1β, and (E) IL‐1α in the urine at 4 h of reperfusion in the control and anakinra groups measured by ELISA. There was a significant reduction in the level of IL‐6 in the anakinra group (*p* = 0.009).

Proximal tubular epithelial cells are particularly susceptible to ischemic injury and this led to high levels of inflammatory cytokines secreted in the urine. There was a significant reduction in urinary IL‐6 levels in the anakinra group compared to controls (*p* = 0.009; Figure 5A). There were no statistically significant differences between urinary levels of IL‐1α, TNFα, IL‐8, and IL‐10 in anakinra‐treated and control kidneys (Figure 5B–E; *p* > 0.05). Some of the samples were below the threshold and therefore the data are not presented. The number per group is noted in the figure legend.

### 3.5 Anakinra did not reduce apoptosis in the renal cortex during NMP

**FIGURE 6:**
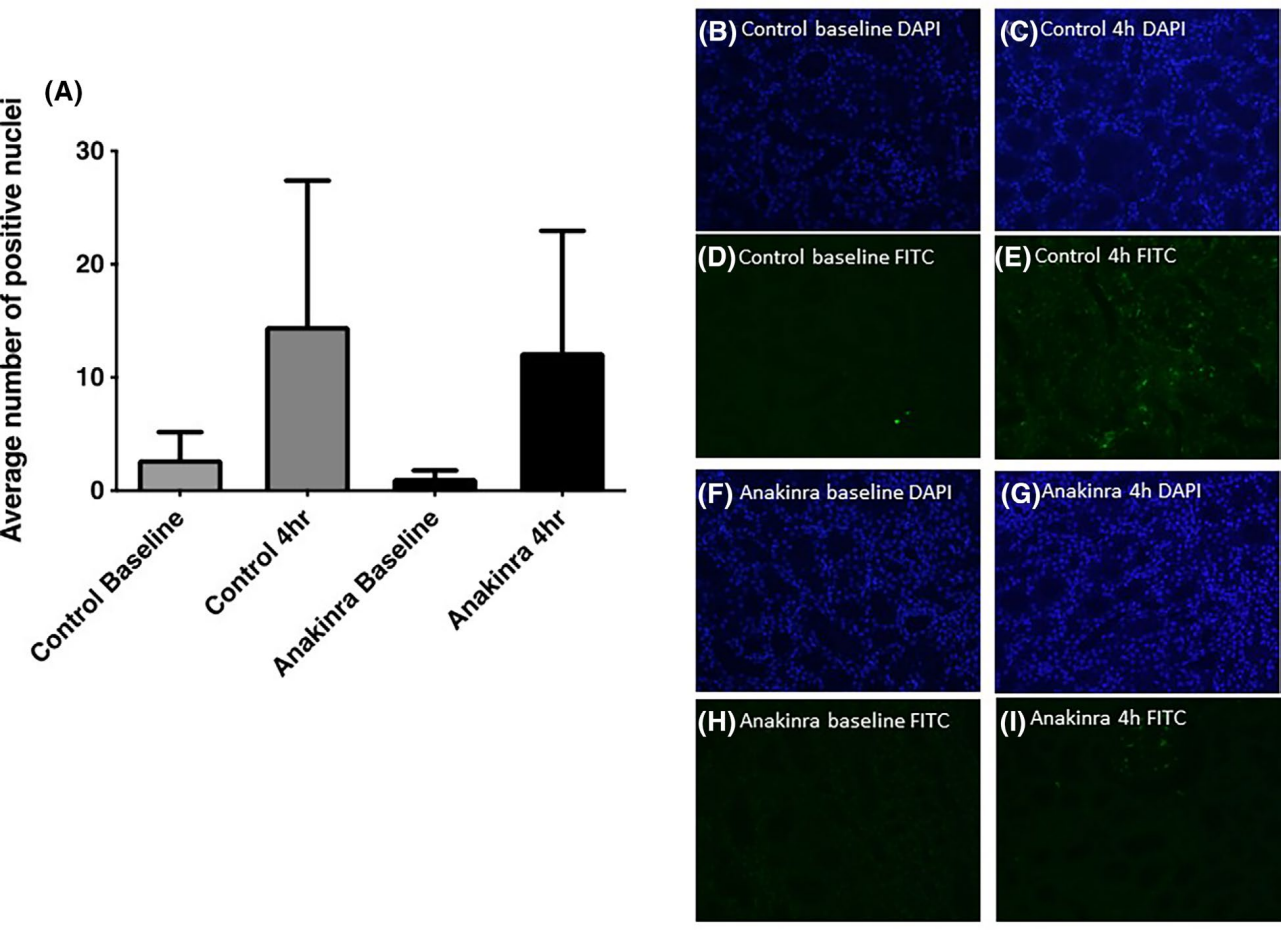
Sections of paraffin‐embedded tissue were stained using the TUNEL technique and visualized using a fluorescence microscope. Ten different fields of view at ×40 magnification images were taken in DAPI and FITC wavelength for each field of view. The mean number of positive cells was counted in the anakinra and control group in the in situ (baseline) and 4 h samples. (A) Anakinra did not significantly reduce the number of positive apoptotic cells (*p* = 0.935). (B, C) Control group DAPI baseline and 4 h biopsies, (D, E) control group FITC baseline and 4 h biopsies, (F, G) anakinra group DAPI baseline and 4 h biopsies, (H, I) anakinra FITC baseline and 4 h biopsies. [Color figure can be viewed at wileyonlinelibrary.com]

Ligation of the IL‐1R1 results in the activation of various apoptosis‐associated transcription factors including NF‐κB and MyD88. The number of renal cortical apoptotic cells, measured by TUNEL staining, was relatively low in all kidneys but increased significantly during NMP. Anakinra did not significantly reduce the number of apoptotic cells (*p* = 0.935; Figure 6).

## 4 DISCUSSION

This study shows that following short warm and cold ischemic injury in porcine kidneys, the administration of the recombinant IL‐1 receptor antagonist anakinra at the start of NMP inhibited IL‐1α and IL‐1β activation. This, in turn, reduced the cascade of downstream inflammatory mediators including IL‐6, ICAM‐1, and Fas ligand. These proof‐of‐principle data support the concept that anakinra has a role in the attenuation of renal injury when administered during NMP.

NMP offers significant potential for the future of transplantation. The ability to administer therapies directly to the kidney to reduce injury may lead to improved outcomes and increase organ utilization. In recent years, further insight has been gained into the molecular and biological changes that occur in the kidney during NMP. Global transcriptomics revealed that TNFα signaling via NF‐κB is highly expressed during NMP in kidneys that developed delayed graft function after transplantation.^9^ Therefore, immune response‐related genes are an obvious target for intervention. NF‐κB is a transcription factor that regulates inflammation by increasing the production of pro‐inflammatory cytokines such as IL‐1β and IL‐6. IL‐1 is a pro‐inflammatory cytokine that induces IL‐6 expression, and these two cytokines are co‐expressed at many sites of inflammation. IL‐1 is also known to upregulate CD11/CD18 expression on leukocytes and increase ICAM‐1 expression on endothelial cells.^10^ The administration of anakinra during NMP reduced the expression of ICAM‐1 at the transcript level, and this could have a beneficial effect in lessening leukocyte activation and adhesion to renal vascular endothelium.

IL‐6 can orchestrate both pro‐ and anti‐inflammatory processes depending on its signaling pathways.^11^, ^12^ IL‐6 is secreted by podocytes, endothelial cells, mesangial cells, and tubular epithelial cells.^13^, ^14^ IL‐1β directly stimulates IL‐6 production.^10^, ^15^, ^16^ Locally, within the kidney, leukocytes can also produce IL‐6 when Toll‐like receptor 4 (TLR4) interacts with high mobility group box 1 protein (HMGB1) released by injured renal cells.^10^ High levels of IL‐6 were detected in the cortical biopsies and in the urine of kidneys in the control group. The administration of anakinra resulted in a significant reduction in levels of IL‐6 transcripts in the renal cortex and in the urine. There was also a significant reduction in the mRNA expression of Fas ligand, which activates apoptosis. In our experimental model only low levels of renal apoptosis were found during NMP and treatment with anakinra did not reduce this already low‐level activity. Taken together, these findings demonstrate that anakinra can reduce the level of inflammation and cell adhesion within the kidney and as such has the potential to ameliorate renal injury.

We started our investigations of the effects of anakinra with a model of relatively mild renal injury that would be reversible. Even so, we have previously shown that the combination of 15 min warm time and 2 h cold storage reduced creatinine clearance when compared to 2 h cold storage after zero warm ischemia.^8^ In this model, although anakinra did not improve creatinine clearance, renal blood flow, or urine output during NMP compared to controls, we demonstrated non‐inferiority, which provides early safety data for the administration of anakinra during NMP as a new delivery system. Our model also allowed us to demonstrate that anakinra exerted significant effects on downstream signaling from the IL‐1 receptor.

In our study, an anakinra bolus of 50 mg was selected to provide a mean circulating plasma concentration of 141 μg/mL. This target level was similar to that in a study in humans examining the optimal plasma concentration of anakinra to achieve penetration into cerebrospinal fluid for inhibition of the acute phase response to stroke.^17^ Other studies in renal transplant recipients report doses of 100 mg administered daily or on alternate days.^18^ Some caution is needed as there is an increased risk of infection due to the additional immunosuppressive therapies. Anakinra is excreted in the urine and when administered systemically has a half‐life of 4–6 h.^19^ In our experimental system, the fall in IL‐1RA levels may be explained by a combination of binding to its targets and urinary excretion, although measurement in urine and tissue would be needed to confirm this.

Systemic administration of anakinra has been studied in patients with chronic renal failure. Patients undergoing hemodialysis commonly have chronic inflammation and this is associated with higher mortality.^20^ In a randomized placebo‐controlled trial, anakinra, administered via the hemodialysis circuit, reduced plasma IL‐6 levels and C‐reactive protein by 25% and 41% of baseline levels respectively.^21^

Anakinra is also safe when administered systemically to renal transplant recipients in combination with standard immunosuppressive therapy.^18^ There are no trials in renal transplant patients to examine the effects of anakinra. However, anakinra has been administered without adverse effects post‐transplant to patients with existing IL‐1‐driven inflammatory conditions. Renal transplantation is inevitably accompanied by ischemia reperfusion injury that may adversely affect early graft function. IL‐1 is released early after transplant revascularization and induces inflammation and apoptosis. In this context, there is some evidence that endogenous IL‐1RA has a protective effect in renal transplant patients. Low serum IL‐1RA levels after renal transplantation have been associated with delayed graft function^22^ and higher urinary IL‐1RA has been associated with improved 1‐year allograft function.^23^

Our study has several limitations. The combination of 15 min of warm ischemia and 2 h of cold storage only produces a mild degree of kidney injury in this ex vivo model.^8^ More severe levels of ischemic injury were not used as they significantly disrupt renal hemodynamics and function.^24^, ^25^ A 4 h period of NMP was chosen as anakinra has a half‐life of 4–6 h, but is a relatively short period to determine outcome. Further studies could investigate the effects of anakinra in more severe ischemic injury and during more prolonged periods of NMP. A constant infusion of anakinra or recirculation of the urine during NMP may ensure a more constant delivery of the drug particularly over longer periods. Furthermore, more information may be gathered from studies in human kidneys offered for research before translation into clinical practice. This would aid in the development of protocols in kidneys with significant renal impairment. Many different kidney NMP protocols have been reported and there remains a lack of consensus on the optimal perfusate conditions. In this study, we used whole blood rather than red cells. Our aim was to replicate a more physiological environment. Nonetheless, recent studies have indicated that there is little difference in terms of metabolic changes or concentrations of inflammatory cytokines when using whole blood or red cells for NMP.^26^, ^27^

Protein expression of IL‐1β was not detectable in the circulating perfusate after 4 h of NMP. This might be explained by the short half‐life in plasma of IL‐1β, which is <5 min, and the timing of expression in kidney tissue, which peaks at 1 h.^28^ IL‐1β permeates the glomerular basement membrane and is almost completely absorbed into the tubular cells. Therefore, earlier sampling might be required for detection.

In conclusion, IL‐1 receptor blockade successfully attenuated the inflammatory response during NMP in porcine kidneys subjected to a mild ischemic insult. This study has provided further insights into the underlying mechanisms of renal injury during NMP and suggests that NMP has potential as a platform for the targeted delivery of treatments to donor kidneys prior to transplantation. Nonetheless, further investigation is needed in human kidneys before application in clinical practice.

## AUTHOR CONTRIBUTIONS

Sarah A. Hosgood and Michael L. Nicholson wrote the manuscript text; Sarah A. Hosgood, Tom Moore, and Alex Walker carried out the experiments, analyzed the data, and prepared the figures. All authors reviewed the manuscript.

## CONFLICT OF INTEREST STATEMENT

The authors declare no conflicts of interest.

## Supporting information


**Table S1.**.
