# Supplementary material for: IL‐1 receptor antagonist anakinra downregulates inflammatory cytokines during renal normothermic machine perfusion: Preliminary results
Source: Artif Organs. 2024 Nov 20;49(3):451–9. doi: 10.1111/aor.14909 (PMC11848953; doi:10.1111/aor.14909)
Supplement: Supplementary file 1 — Table S1.. [file AOR-49-451-s001.docx]

**Supplementary Material**

**Supplementary Table 1.** qPCR primers

| Gene | NCBI accession number | Primer sequences |
| --- | --- | --- |
| Angiopoietin 1 | [NM_213959.1](https://www.ncbi.nlm.nih.gov/entrez/viewer.fcgi?db=nucleotide&id=47522747) | TCCAGGAACTGAAAAAGCAATTGAA |
|  |  | AGTAAAACACCTTCTTTAGTGCAAA |
| Fas ligand | [NM_213806.1](https://www.ncbi.nlm.nih.gov/entrez/viewer.fcgi?db=nucleotide&id=47523227) | CCTTGGTCTCTGGGGTGAAG |
|  |  | CCCCGGAAGTACACTTTGGA |
| HPRT1 | [NM_001032376.2](https://www.ncbi.nlm.nih.gov/entrez/viewer.fcgi?db=nucleotide&id=119310177) | CCAGCGTCGTGATTAGTGAT |
|  |  | ATCTCGAGCAAGCCGTTCAG |
| ICAM-1 | NM_213816.1 | TCAATGTGGCCCCTAAACACC |
|  |  | GTCTCTAGGCCAAAGCTGGT |
| IL-1β | NM_214055.1 | CCTTGAAACGTGCAATGATGACT |
|  |  | GCCAGCCAGCACTAGAGATT |
| IL-6 | NM_001252429.1 | GGGTTCAATCAGGAGACCTGC |
|  |  | CGGCCTCGACATTTCCCTTA |
| IL-8 | NM_213867.1 | AGAGTGGACCCCACTGTGAA |
|  |  | TGTTGTTGCTTCTCAGTTCTCTT |

**Supplementary Table 2.** PCR thermocycler conditions

|  | Temperature (°C) | Time (seconds) |
| --- | --- | --- |
| Segment 1  1 cycle | 95 | 600 |
| Segment 2  40 cycles | 95 | 30 |
|  | 60 | 60 |
| Segment 3  1 cycle | 95 | 60 |
|  | 60 | 30 |
|  | 95 | 30 |
